# Enhancing the efficacy of integrative improvisational music therapy in the treatment of depression: study protocol for a randomised controlled trial

**DOI:** 10.1186/s13063-019-3323-6

**Published:** 2019-04-29

**Authors:** Jaakko Erkkilä, Olivier Brabant, Suvi Saarikallio, Esa Ala-Ruona, Martin Hartmann, Nerdinga Letulė, Monika Geretsegger, Christian Gold

**Affiliations:** 1 Music Therapy Clinic for Training and Research, Department of Music, Art and Culture Studies, University of Jyväskylä, PO Box 35 (M), FI-40012 Jyväskylä, Finland; 2 Uni Research Health, Grieg Academy Music Therapy Research Centre, Postboks 7800, 5020 Bergen, Norway

**Keywords:** Depression, Anxiety, Music therapy, Clinical improvisation, Resonance frequency breathing, Homework, Integrative psychotherapy

## Abstract

**Background:**

Depression is among the leading causes of disability worldwide. Not all people with depression respond adequately to standard treatments. An innovative therapy that has shown promising results in controlled trials is music therapy. Based on a previous trial that suggested beneficial effects of integrative improvisational music therapy (IIMT) on short and medium-term depression symptoms as well as anxiety and functioning, this trial aims to determine potential mechanisms of and improvements in its effects by examining specific variations of IIMT.

**Methods/design:**

A 2 × 2 factorial randomised controlled trial will be carried out at a single centre in Finland involving 68 adults with a diagnosis of depression (F32 or F33 in International Statistical Classification of Diseases and Related Health Problems 10th revision). All participants will receive 6 weeks of bi-weekly IIMT, where they are invited to improvise music and reflect on those improvisations with a music therapist in a one-to-one setting. Potential enhancements to IIMT will include: home-based listening to recorded improvisations (LH) from IIMT sessions to facilitate integration of therapeutic processing into daily life; and resonance frequency breathing (RFB), a breathing exercise at the beginning of each session to facilitate emotional expression and processing. Participants will be randomised in a 1:1:1:1 ratio into each combination (IIMT alone or with one or both enhancements). The primary outcome is depressive symptoms measured by the Montgomery–Åsberg Depression Rating Scale (MADRS) at 6 weeks. Secondary outcomes are depressive symptoms at 6 months; anxiety, quality of life, and functioning at 6 weeks and 6 months; and adverse events. Secondary underlying mechanisms/process variables are self-rated momentary depression level before every IIMT session; and homework compliance in IIMT + LH. Statistical analyses involve an intention-to-treat approach, using a linear mixed-effects model examining the main effects (LH vs no LH; RFB vs no RFB) and interaction effects (LH × RFB).

**Discussion:**

This trial will contribute to understanding the mechanisms of IIMT and may further enhance the effectiveness of an intervention that was previously shown to be superior to standard care alone for adults with depression.

**Trial registration:**

ISRCTN11618310. Registered on 26 January 2018.

## Background

Depression is one of the psychiatric disorders that can effectively be treated through music therapy [1]. In previous music therapy trials for depression, diverse clinical techniques relying on multiple methods have been employed. However, Erkkilä et al. [2] showed that applying only one clinical technique—in that case clinical improvisation—was effective as well. Thus, the previous evidence does not exclusively speak for multimethod clinical approaches. Along with the increased evidence favouring music therapy as an effective form of treatment for depression, there is a need to take the next step—to identify the active ingredients of music therapy, meaning the elements that enhance patient outcomes [3]. This is important in particular because although Erkkilä et al. [2] found that many participants responded to usual improvisational music therapy, proving it to be effective on the group level, there were also those clients who did not respond or who responded less than others. This finding raises the question of whether the efficacy of clinical improvisation can be further enhanced in order to increase the response rate.

### Integrative improvisational music therapy

One of the specific music therapy techniques that has been demonstrated to be effective in the treatment of depression is integrative improvisational music therapy (IIMT). IIMT is a model that is built on a versatile theoretical background. It was originally rooted in psychoanalytical ideas about the meaning of music [4, 5], in improvisational music therapy used in a psychodynamic context [6], as well as in psychodynamic psychotherapy [7]. This legacy is still important for IIMT. However, along with the development of integrative psychotherapy [8], which emphasises the needs of the client in changing situations instead of closely following certain, tightly framed theoretical or practical guidelines and rules, it became natural to definitionally update our model and better describe the flexible clinical thinking and practice associated with it. Therefore, in addition to being psychodynamic at its core, IIMT also involves supportive psychotherapy, which integrates psychodynamic, cognitive-behavioural, and interpersonal views [9] as well as resource-oriented music therapy [10], which, among other things, redefines the nature of the therapeutic relationship.

The unique and essential component [11] of IIMT is the clinical improvisation. The sessions consist of a combination of improvising in a client–therapist dyad and discussing the experiences triggered by the improvisations [12]. As a form of non-verbal expression and interaction, clinical improvisations lead to the emergence of abstract and unshaped mental contents that are often accompanied by emotional content. A clinical improvisation typically triggers emotions, emotional memories, images, metaphors, and associations, which can be further processed in the verbal domain [13]. In essence, musical improvisation serves as the experiential foundation for meaning formation.

Improvisational music therapy is suitable for basically all client groups without the need for specific musical training, and can be successfully applied even when one’s ability for verbal expression is limited [13, 14]. Any adult with normally developed cognitive skills and abstract thinking is able to connect symbolic and experience-based mental contents to their musical expression in clinical improvisations. They are also able to verbalise, in other words to further process, their music-based experiences and to interpret these experiences in the light of their current life situation [15].

### Processing of difficult emotions as an active ingredient

Our experience in working with adults with depression has shown that clinical improvisations often trigger anxious, negative emotions and experiences related to the illness. Furthermore, the clients appear to improve through IIMT particularly if dealing with these challenging emotions. We therefore started hypothesising that there is something in improvisational music therapy which especially facilitates the emergence of traumatic material and negative emotions, yet in a way that is tolerable to the client. When looking at the literature, a recurrent finding is that emotional processing plays a key role in achieving a positive therapeutic outcome. Increased emotional processing generally leads to better outcomes, while avoidance and suppression worsen the conditions [16, 17]. Expressing, accepting, and transforming the unwanted thoughts and related negative emotions is essential for psychotherapy to be successful; as Hunt [18] pertinently summarised, “the only way out is through”.

Several definitions of emotional processing have been proposed [19]. However, based on the ideas developed by Greenberg and Pascual-Leone [20], at least the following appear to be core ingredients: certain emotions are activated (arousal); the client is able to acknowledge, allow, and tolerate these emotions by finding a middle ground between avoidance and over-engagement (regulation); and the emotional experience is explored and reflected upon, for example, through symbolisation and meaning-making. This definition would apply to emotional processing in any form of emotion-focused or experiential therapy, including music therapy, whose mode of action is essentially emotion based [21].

### Music listening as promoter of emotional processing

The special potential of music to facilitate the processing of complex negative emotions is supported by music psychology research. Music listening has been shown to induce complex emotional experiences [22, 23], and to support elaborate self-regulatory processing of sometimes difficult, personal emotional states in everyday life [24]. Furthermore, music is often characterised—somewhat paradoxically—by pleasurable experiences of difficult emotions, such as sadness [25]. It has been argued that the enjoyment of sadness in music is explained by the capacity of music to allow simultaneous absorption, yet dissociation, from the affective content [26, 27]. Music characteristically allows access to various degrees of emotional nuances and intensities with simultaneous self-reflection, detachment, and elaborative awareness of these emotional nuances [28], supporting both experience and conceptual awareness of feelings [29]. Overall, the findings support the idea that music holds special potential for the self-reflective processing of complex, difficult, and even painful emotions.

However, music listening patterns have been shown to differ in terms of how adaptive and health beneficial they are, and depressed individuals in particular are inclined towards using the unhealthy/maladaptive patterns [27, 30–33]. Depressed individuals thus are generally less able to benefit from the affordances of music, but pioneering work indicates that music therapy interventions can help the clients to learn more health-beneficial ways of using music in their own daily lives [34]. When the music listening experience is combined with the therapy process and targeted towards the clinical improvisations created during therapy, it becomes possible to focus more on adaptive patterns (e.g. reflection instead of rumination). The listening-back experiences can then further be discussed and processed with the therapist during the sessions. This is expected to result in an upward spiral of adopting resources for reflecting on personal experiences in an adaptive manner, with the possibility of transferring such accomplishments in therapy to the client’s everyday life. This could potentially be highly relevant, particularly in terms of sustaining the long-term impact of the therapy.

In sum, we propose that combining music making (improvising) and music listening (listening back to the recorded clinical improvisation as homework) has mutually complementary effects on therapeutic processing. Listening back to the improvisations may particularly promote the self-reflective processing of the emotions and personal experiences expressed through the improvisation. We therefore aim to assess the combined effect of improvisation and listening activities.

### Resonance frequency breathing as a promoter of emotional processing

Starting the therapy sessions with a specific type of slow breathing is another avenue we wish to explore. The purpose of this preliminary intervention would be to relax the clients and prepare them for deeper therapeutic work. Indeed, one well-known benefit of slow breathing is to reduce autonomic arousal, which can be observed in pranayama yoga for example [35, 36]. It should be noted that the idea to start psychotherapy sessions with a breathing intervention has already been proposed by other authors [37, 38]. However, this suggestion has been very little implemented. Usually, when a breathing intervention is systematically used for emotional or psychological disorders, it is in the form of a stand-alone intervention, with no integration into another, primary psychotherapeutic method.

The breathing intervention we will be using is known, among other names, as resonance frequency breathing (RFB), which is the core component of a method called heart rate variability biofeedback (HRVB). The idea behind HRVB is to help clients breathe at their resonance frequency, through which they achieve a state known as psychophysiological coherence [39]. This state of resonance or coherence has two main characteristics, clearly detectable on the physiological level: the amplitude of heart rate oscillations is maximised, while the heart, respiratory, and blood pressure rhythms become highly synchronised [40]. This results in an immediate shift of the autonomic nervous system towards parasympathetic (rest-and-digest) dominance, leading to a state of calm alertness. It should be noted that the optimal breathing speed under which resonance is achieved is different from person to person. However, we know from previous studies that for an adult the optimal speed is located somewhere around 6 breaths/min [41].

Regarding the potential benefits of using RFB in conjunction with IIMT, we have already collected some promising evidence through two single-case experimental studies involving a healthy client [42] and a client diagnosed with anxiety disorder [43]. The results indicate that RFB might be an adaptive intervention, capable of both supporting and challenging the client, depending on the therapy phase and the client’s current emotional needs. In the light of these exploratory results, we hypothesise that RFB will favour the emergence of difficult, negative emotions during therapy, while at the same time improving the client’s tolerance for and ability to process such emotions. Should this indeed be the case, we expect that the addition of RFB to IIMT will lead to better therapeutic outcomes, with better emotional processing being the mediator.

### Aims of the study

Self-reflective processing of negative emotions may well be a core ingredient in explaining the effectiveness of clinical improvisations and IIMT in general. Therefore, this study is designed to apply methods for enhancing this type of processing and testing their impact on the outcomes of IIMT in the treatment of depression. Two different types of methods will be added to create the experimental conditions of the trial—listening back to improvisations (LH) and resonance frequency breathing (RFB). The study’s main hypothesis is that the addition of LH or RFB will have a favourable effect on the therapy outcome. In addition, we will examine the internal mechanisms of IIMT in order to gain a better understanding of how they contribute to recovery. Lastly, we will examine whether IIMT has certain unique or exceptional characteristics that support its use in the treatment of depression in general.

## Method

### Study design

#### Participants, recruitment, and the site

Participants will be recruited in central Finland through announcements placed in local newspapers. Our experience has shown that this approach is more effective than targeting only mental health clinics and psychiatric polyclinics. We will repeat the announcements as often as needed for reaching the recruitment target. The trial will be conducted at the Music Therapy Clinic for Training and Research, Department of Music, Art and Culture Studies, University of Jyväskylä, Finland.

#### Inclusion and exclusion criteria

As inclusion criteria, the participants must be between 18 and 55 years of age and have depression as their primary diagnosis (categories F32 and F33 of the ICD-10), assessed by the Montgomery–Åsberg Depression Rating Scale (MADRS). In addition, if it is diagnosed alongside the depression, participants with anxiety will also be included, as both pathologies present a high level of comorbidity. Musical skills or any form of musical background are not required; their presence, however, does not constitute a reason for exclusion.

The exclusion criteria are psychosis, combined psychiatric disorders in which depression cannot be defined as the primary disorder, acute and severe substance misuse, and depression severity preventing the clients from participating in the measurements or engaging in verbal conversation. Furthermore, participants must be able to complete written questionnaires. Therefore, people with insufficient knowledge of the Finnish language or who are otherwise unable to complete such a questionnaire will be excluded.

#### Trial design and hypotheses

The study is a randomised 2 × 2 factorial trial, in which the conditions are derived from either the presence or non-presence of listening homework (LH) and resonance frequency breathing (RFB). The design is presented in Table 1. All groups receive IIMT. In addition, one treatment group receives LH as an additional component, the second treatment group receives RFB as an additional component, and the third treatment group receives both of these as an additional component.

**Table 1:** Trial design: 2 × 2 factorial

Presence of additions	LH present (*n* = 34)	LH not present (*n* = 34)
RFB present (*n* = 34)	IIMT + LH + RFB (*n* = 17)	IIMT + RFB (*n* = 17)
RFB not present (*n* = 34)	IIMT + LH (*n* = 17)	IIMT (*n* = 17)

*IIMT* integrative improvisational music therapy, *LH* listening homework, *RFB* resonance frequency breathing

A decrease in depression scores is expected in all groups, as IIMT as such is already known to be effective in the treatment of depression [2]. In this study, we further hypothesise a significant main effect of both additional interventions:

- There will be a significant main effect of LH.
- There will be a significant main effect of RFB.

Based on this, we expect that each treatment group will show an increased improvement in comparison to the IIMT-only group. The greatest improvement in depression is likely to be observed in the group receiving both additional treatment components (IIMT + LH + RFB), but the investigation of the potential interaction effect of the additional treatments is kept exploratory. We also hypothesise faster recovery—an earlier drop in depression scores—in the enhanced treatment conditions compared to the IIMT-only condition.

#### Randomisation and sample size

**Fig. 1:**
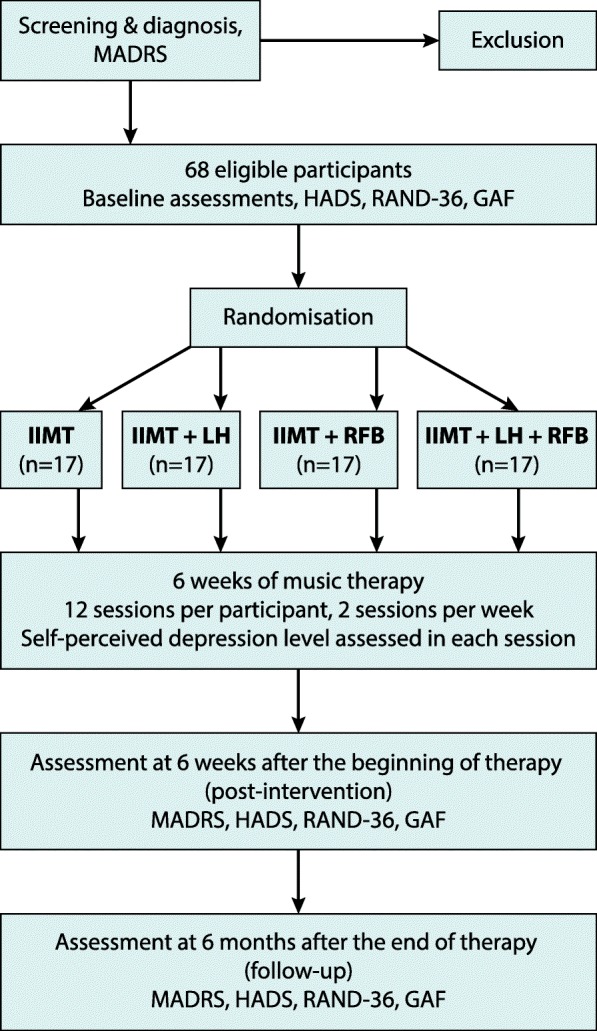
RCT flow chart with outcome measures. GAF Global Assessment of Functioning, HADS-A Hospital Anxiety and Depression Scale—anxiety subscale, IIMT integrative improvisational music therapy, LH listening homework, MADRS Montgomery-Åsberg Depression Scale, RAND-36 quality-of-life survey, RFB resonance frequency breathing

Figure 1 shows how participants eligible for the randomised controlled trial (RCT) flow through the study from recruitment to follow-up. After screening and diagnosis, 68 participants will continue to take part in the study. Following a baseline assessment they will be randomly assigned to one of the four combinations of conditions (*n* = 34 in each condition; *n* = 17 in each combination).

An external expert who has no direct contact with the patients will conduct a computerised randomisation procedure. We will employ block randomisation with randomly varying block sizes of 4 and 8. The allocation list will be saved on a password-protected repository with access only by the PI and study coordinator. To ensure allocation concealment, the randomisation will be conducted at another site (Uni Research, Bergen, Norway). As a result, the assessor, the therapists, and the participants will not be able to know the group allocation in advance. Only the outcome assessor will remain blinded to the group allocation throughout the trial. At the time of assessments, participants will be reminded not to reveal their allocation to the assessor. As far as the PI is concerned, he is blinded from the randomisation and the clients’ conditions in general, but because he is also one of the clinicians and participates in supervision he will get to know the condition of some clients. However, the PI is not involved in the client assessments or data entry in any way.

A power calculation was carried out for the primary outcome (depressive symptoms measured by the MADRS), based on the following assumptions.

Prior reviews of music therapy used in the treatment of depression have reported large effect sizes of music therapy compared to standard care. A recent meta-analysis [1], which included nine studies and involves a total of 421 participants, examined the short-term effect of music therapy for depression for primary outcomes. These authors found moderate quality evidence favouring music therapy and treatment as usual (TAU) over TAU alone for both clinician-rated depressive symptoms (SMD – 0.98, 95% CI – 1.69 to − 0.27, three RCTs, one CCT, *n* = 219) and patient-reported depressive symptoms (SMD – 0.85, 95% CI – 1.37 to − 0.34, three RCTs, one CCT, *n* = 142). Regarding IIMT specifically, our previous study found a medium effect size of *d* = 0.65 [2] based on the traditional two-arm design (intervention vs control).

As regards our additional components, a meta-analysis of the effect of HRVB training on stress and anxiety [44] reported large effect sizes with both the pre–post within-group effect size and the between-group analysis comparing HRVB to a control condition yielding Hedges’ *g* of around 0.8. A meta-analysis of the effects of homework assignments on treatment outcome in cognitive and behavioural therapies [45] reported a pooled ES of *d* = 0.48 favouring homework.

With the target sample size already reported (34 in each main effect), a two-sided *t* test at the 5% significance level will have 50% power to detect an effect size of 0.48 (LH) and a 90% power to detect an effect size of 0.8 (RFB). We provide these numbers with reservations and for information only, since these meta-analyses about homework and HRVB were not conducted in an identical setting to our trial. First of all, HRVB was studied as a stand-alone intervention, whereas we are using it in combination with another method. Second, the type of homework used in IIMT (listening back to self-created music improvisations) is arguably quite different from the homework usually given in cognitive behavioural therapies (e.g. exposure, thought diaries, or practice of social skills). Therefore, we acknowledge that the main effects of RFB and LH may be different from those found in the literature. Consequently, our study remains exploratory, and will only provide evidence for possible strong effects of the added components.

The minimum clinically important difference (MCID) for the Montgomery–Åsberg Depression Rating Scale (MADRS) has been reported to vary between 1.6 and 1.9-point changes from baseline when using a distribution-based method [46]. For anchor-based methods, prior studies report a remission cut-off point as a total score of < 9 points after treatment, or that a score of ≤ 5 equals complete or symptom-free remission and ≤ 11 equals remission, and that a decrease in 39% from baseline corresponds to “much improved” on the Clinical Global Impressions Scale. A mean difference of 1.6–1.9 points, with a common SD of around 7 [2], corresponds to an effect size of around 0.25, which is much lower than the effect size for which this study is powered. Thus, the study will not be able to reliably detect an MCID, but will be powered to detect an effect size similar to those found in previous related studies, as described earlier.

### Interventions

#### Overall intervention structure

The intervention phase consists of 6 weeks of music therapy, composed of 12 sessions, with two sessions per week (see Fig. 1). The length of each session is 60 min. The trial will not interfere with any concomitant treatments the clients may already be receiving or that may be required during the trial. The clients are informed of their right to leave the trial at any point without the need to give any justification. The content and working principles concerning IIMT and the additional components (LH and RFB) are described in the following sections.

#### Integrative improvisational music therapy

Integrative improvisational music therapy (IIMT) is used as the therapy model for all of the intervention conditions. IIMT is a model developed at our site [12] and successfully employed in our previous RCT for depression [2]. As described in the Introduction, the non-verbal expression and interaction of clinical improvisation typically leads to the emergence of abstract and unshaped mental contents, often accompanied by emotional loading. The evoked emotions, emotional memories, images, metaphors, and associations can then be further processed on the verbal domain, which creates the possibility for meaning formation [12].

Similar to the prior RCT, IIMT in the current RCT will consist of: 5–10 min of initial discussion; improvising in a client–therapist dyad with easily approachable instruments (drums and electric piano); and discussing the experiences triggered by the improvisations [12]. The number and length of the improvisations can vary depending on the client’s situation and the phase of the therapy process. Occasionally, clients may have difficulties verbalising their experiences, in which case the interaction may rely more on music. It may also be the other way around, whereby a client has a strong need to verbalise his/her musical experiences, thus creating less room for music. In other words, although based on a single music therapy technique (clinical improvisation), IIMT offers flexibility as well.

#### Added component: listening homework

LH will be conducted as an activity carried out outside the therapy context, in the client’s own time. This activity can be described as homework, which is a known practice particularly in cognitive and behavioural psychotherapies. In that field, two meta-analyses [45, 47] have been conducted, showing that homework compliance is significantly related to the therapeutic outcome, with the effect size varying from small to medium. What is important to our study is the fact that, according to the findings from cognitive and behavioural psychotherapies, the clients with depression particularly seem to benefit from homework.

In our study, the music listening homework specifically aims to foster the emotional processing associated with the clinical themes and experiences associated with the music improvisations created during the sessions. As described in the Introduction, music psychology research has widely evidenced the potential of music listening to serve as a forum for self-referential (and even auto-therapeutic) processing of emotional content.

In practice, the clinical improvisations are recorded during the music therapy sessions. A specially developed computer application allows each client to have personal access to his/her recorded improvisations on the university server. Clients will be able to listen to the music whenever they want and as many times as they want throughout the therapy process. This application also monitors the listening behaviour (frequency and duration), thus allowing us to evaluate the clients’ adherence to the task. While clients have access to all of their improvisations throughout the therapy process, however, after each session the clients are encouraged to listen to the clinical improvisations of that session, and the therapist may recommend particular improvisations. Experiences of listening back to improvisations are discussed and reflected upon with the therapist during the subsequent therapy session(s).

#### Added component: resonance frequency breathing

RFB is the second tool we wish to use for promoting self-reflective emotional processing. RFB, in its stand-alone form, has specifically been shown to be efficacious in reducing stress and anxiety [44]. Moreover, two pilot and open-label studies have led to positive results with depression [48, 49]. However, little is known about the effects of RFB when used in combination with improvisational music therapy. Our own exploratory studies indicate that clients of the RFB conditions might be able to open up more and express more therapeutically relevant emotions during the sessions, both on the verbal and musical levels, which, in turn, should lead to better therapy outcomes.

Regarding the implementation of RFB, we will not be using any biofeedback apparatus as would be done with HRVB. Instead, we will be determining each participant’s resonance frequency beforehand, using the protocol described by Lehrer [50]. This alternative approach to HRVB is possible because the resonance frequency has been shown to be stable in adults [41]. Once their resonance frequency has been established, a tablet with a breathing app set at the correct speed will be used for cueing the participants during RFB. Furthermore, we will be using an inhalation/exhalation ratio of 40/60, because longer exhalations are known to support parasympathetic activation [51]. RFB will be performed for 10 min at the beginning of each session, with the participants sitting in an upright position and the tablet placed in front of them.

#### Treatment fidelity and training the clinicians

The intervention will be conducted through 11 clinically trained music therapists (according to Finnish music therapy training standards) who have a long experience in music therapy work. Most have also participated in our previous RCT [2] and have extensive prior experience of using the IIMT model in their practice. Additionally, four of the recruited music therapists are also qualified psychotherapists.

All of the clinicians will go through intensive training to deepen their knowledge of the working principles of IIMT as well as the differing requirements of the four treatment conditions. In order to control for the role of the therapist in the groups’ outcomes, each clinician will be conducting therapy in all of the four conditions of the study.

The training period consists of 24 h of training in the IIMT model (eight weekly training sessions, 3 h each). In the training, the clinicians are presented with the idea, design, and content of the No Pain No Gain (NPNG) project, and are familiarised with the research measures and data collection principles they need to master for the RCT. The clinicians are, for instance, responsible for administering the continuous assessment of the perceived depression level, collecting session feedback, and implementing the audio-recording protocol for the home listening task.

First and foremost, it is crucial that the clinicians develop a common understanding of the theoretical and practical aspects of IIMT. To this end, the training includes lectures on IIMT theory accompanied by slide shows, articles, handouts, and video demonstrations that will remain available to the clinicians throughout the project. The improvisational techniques of IIMT will be demonstrated in every meeting, as well as the ways of creating a therapist–client relationship through the combination of clinical improvisation and talking. To increase the authenticity of the training, outside volunteers will be playing the client’s role.

For the clinical work phase, a compendium has been created from which the clinicians are advised to check the essential elements and contents of the model when needed. In addition, the members of the NPNG research group are available during the trial for any urgent needs, questions, or problems that might arise on a daily basis.

Throughout the NPNG project, the clinicians meet together every 2 weeks for group supervision. In these meetings the clinicians are supposed to focus on their own sessions and raise the most challenging issues encountered in their clinical work, with the aim of discussing and solving them. The supervision meetings are peer based, meaning there is no specific or external supervisor in charge of them. Instead, the therapists are all contributing according to their own expertise, four of them being trained supervisors through their psychotherapy qualification. Video and audio material from the sessions will be available for supervision purposes. In addition to peer-based supervision work, external supervisors/experts will be invited whenever the situation requires. The supervision sessions are also an opportunity to remind the therapists about the principles of the treatment model.

### Assessment

**Fig. 2:**
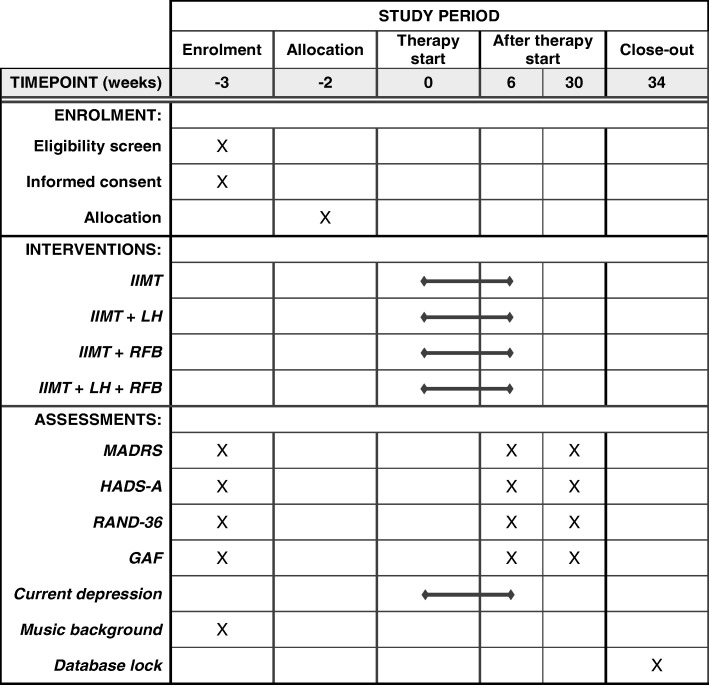
Schedule of enrolment, interventions, and assessments. GAF Global Assessment of Functioning, HADS-A Hospital Anxiety and Depression Scale—anxiety subscale, IIMT integrative improvisational music therapy, LH listening homework, MADRS Montgomery–Åsberg Depression Rating Scale, RAND-36 quality-of-life survey, RFB resonance frequency breathing

The outcome measures of the RCT are described in the following. Overall, there are three measurement points: during the recruitment of the participants (baseline); 6 weeks after the beginning of the intervention (post intervention); and 6 months after the beginning of the intervention (follow-up). Our time point of primary interest is the post intervention, the closest point to the end of the intervention. The timing for administering each outcome measure is shown in Fig. 2: the speed of improvement concerning the clients’ self-perceived depression level throughout the process is assessed at every session, while all other outcome measures are administered at the three measurement points. In addition to the outcome measures, demographics including gender, age, and socio-economic background will be inquired at the beginning of the intervention. The baseline assessment will also include questions on music background (holding a degree in music, amount and type of music engagement) to provide an overview of sample characteristics. All assessments will be conducted by a medical expert specialised in psychiatric evaluation. The assessor is responsible for contacting and reminding the clients of assessment meetings (pre, post, and follow-up). Our previous trial showed that when treatment is offered for free to the client, the dropout rate remains low. Therefore, we have not developed additional client retention strategies.

#### Primary outcome

The Montgomery–Åsberg Depression Rating Scale (MADRS) will be used to assess the severity of depressive symptoms, serving as the primary outcome measure of the study. The MADRS consists of 10 questions rated on a scale from 0 to 6, and the rating is based on an interview conducted by a clinician. At the beginning of the study, MADRS will be administered as part of the screening process, thus serving also as the source for determining eligibility for the study.

#### Secondary outcomes

The Hospital Anxiety and Depression Scale (HADS) will be used to assess anxiety. The HADS is a widely used and well-established self-report questionnaire for assessing anxiety and depression in a non-psychiatric adult population. In the present study, only the anxiety subscale will be used (HADS-A). This consists of seven items scored from 0 to 3, with higher scores indicating higher levels of anxiety. The Finnish version of the HADS has been found to be reliable and internally consistent, with a Cronbach’s alpha of 0.83 [52].

Quality of life will be assessed with the RAND-36. The RAND-36 is a health survey with 36 items that focus on eight domains related to quality of life: physical functioning, bodily pain, role limitations due to physical health problems, role limitations due to personal or emotional problems, emotional well-being, social functioning, vitality, and general health perceptions. It is often used to evaluate someone’s quality of life. The RAND-36 has been officially translated into Finnish, and the reliability of the localised version was determined to be adequate, each subscale having a Cronbach’s alpha above 0.80 [53].

The Global Assessment of Functioning (GAF) is a scale used to assess how mental health symptoms are affecting a person’s daily life and general functioning. The GAF is clinician rated and administered through an interview. The scoring ranges from 0 to 100, and the scale is subdivided into 10 sections. The GAF has been officially translated into Finnish by the Social Insurance Institution of Finland (KELA) and is routinely used by health care providers.

A one-item rating of current depression severity will be used as an additional secondary outcome measure for tracking the trajectory of change concerning the client’s self-perceived depression levels. At the beginning of each session, the clinician will ask the participant to describe his or her current level of depression on a scale from 0 = *not at all depressed* to 10 = *extremely depressed*. The clinician stores the numeric answer. This allows us to follow the clients’ self-perceived depression levels throughout the therapy process and assess the speed of recovery.

#### Safety assessments

Based on previous research evidence it can be concluded that a majority of the participants should benefit from the intervention. The treatment model and the measurements associated with it are all non-invasive. Some aspects of the RFB intervention may cause momentary uncomfortable experiences to some of the participants. These will be minimised by appropriate information, by sensitively listening to the experiences of the participants, and by always ensuring that a participant is capable and willing to participate in the activities in question. Possible concomitant care and interventions are not prohibited during the trial.

The intervention (IIMT) may temporarily trigger emotions and experiences perceived as uncomfortable, for instance negative emotions, associations, and feelings related to a certain therapeutic theme, or one’s illness in general. Based on our experience, however, IIMT is not different from typical psychotherapeutic processes where difficult emotions, worries, fear, and feelings of insecurity are temporarily experienced; even temporary worsening of the depression is possible. Due to the relative shortness of the process (12 sessions), the therapists’ training emphasises how to handle the clients’ possible negative experiences within the sessions. For instance, the recommended practice is to try to reduce the emotional intensity of the session towards the end of it so that the client would leave the therapy room as peaceful and calm as possible. These issues are emphasised both in the training of the clinicians before the RCT and in the supervision during the RCT. When ending the therapy session—and, in particular, when ending the whole therapy process—the therapists are instructed to pay particular attention to the client’s overall, emotional well-being.

For the participants, there are two main contexts in which they can give additional feedback regarding the treatment, the first being an electronic questionnaire administered at the end of every session. The questionnaire covers aspects related to the session’s content and impact, as well as the client’s emotional experiences. The second possibility is the post-treatment assessment, where the assessor, in addition to administering the outcome measures, asks the clients for general feedback regarding the treatment and their participation in the trial.

Our previous RCT [2] had a low incidence of adverse events and we expect this rate to remain low in the new RCT. However, in this trial over twice as many clients will receive music therapy and it is possible that the total amount of treatment-related adverse events will be larger than in our previous trial. In addition, the newly introduced elements (RFB and LH) may increase the risk of adverse events. Taking these points into account, we expect to encounter the following categories of adverse events: treatment-model related (some clients may experience IIMT as inappropriate and unpleasant for them, which may cause frustration and/or anxiety); client related (despite the careful initial assessment to ensure meeting of the inclusion criteria, some participants with additional psychiatric disorders may be enrolled; this may lead to unexpected challenges and it is possible that a client’s condition will worsen); and intensity related (bi-weekly sessions and the presence of added treatment components may increase the depth and intensity of psychological/emotional processing, which might cause participants to feel anxious and could lead to a possible worsening of a client’s condition, at least temporarily).

For reporting the adverse events, a form will be created including the description of the incident plus additional information such as date, participant code, time, site, and the name of the staff person (if applicable). A procedure for addressing the report to the right place will be created.

Because of their existing diagnosis, we can assume that the majority of the participants are already being treated inside the mental health care system. For instance, in our previous study, about 70% of the participants took medication for depression before and during the RCT, and were in more or less frequent contact with mental health services. The clinicians will be advised to discuss the possible need for further therapeutic activities with the client. In particular, if the clients have no previous or current contact with the health care system regarding their depression, or if they are unaware of the treatment possibilities, they will be told about the local treatment practices and possibilities. Despite all the precautions, should a client’s condition worsen and no solution be found between the therapist and the client, the therapist is advised to contact the PI. The PI is responsible for starting appropriate actions, for instance contacting an external expert for remedial actions (e.g. the psychiatrist in charge).

### Statistical analyses and data management

The statistical analyses will follow an intention-to-treat approach, using all available data regardless of whether the treatment was received as intended. Furthermore, in the event of missing outcome data, participants will remain in the analysis if at least one follow-up time point is available. Sensitivity analysis will be conducted with multiple imputation for missing data and as a per-protocol analysis (treatment as received). All outcome measures (MADRS, HADS, RAND-36, GAF, one-item rating of current depression severity) provide continuous data and will be examined for normality before statistical tests are conducted. The main effects of each intervention (LH vs no LH; RFB vs no RFB) and their interaction (LH × RFB) will be tested for all outcome measures, using a repeated-measures linear mixed-effects model with LH and RFB as factors. This analysis strategy has the advantage of making full use of the factorial design, such that all 68 participants are available for each comparison (as presented in Table 1). All tests will use two-sided 5% significance level, with no adjustments for multiplicity; secondary outcomes will therefore be regarded as exploratory. No interim analyses of effects will be conducted.

Most of the data obtained through scales, questionnaires, and forms will be collected electronically. Should the data be obtained through paper forms, they will be immediately converted to electronic data. Data entries are always made by an assessor who is very familiar with the assessment instruments and can thus easily recognise inaccurate data. The assessor will also double-check the data entries. All of the collected data will be stored on secure servers from the University of Jyväskylä, which includes daily backups. Dedicated password-protected folders will be used for different types of data, with access rights granted only to those researchers allowed to handle the given data.

Personal records of the participants, such as names, contact details, and consent forms, are stored in a locked room, with access only by the PI, assessor, and study coordinator. In all other contexts, client codes will be used instead of personal information.

The final dataset will only include pseudonymised data, and will be accessible to all of the researchers involved in the project. We are currently not allowed to make any data available to outsiders through a data repository. We might consider amending our ethical permission in the future so as to share part of the data.

We are planning to publish one main study with the primary and secondary outcomes in a psychiatric journal, as well as several articles focusing on specific sub-questions related to the internal mechanisms of IIMT.

## Discussion

This research builds on the results of our previous RCT about the effect of individual, improvisational music therapy for depression [2] where we showed that the experimental group who received IIMT improved significantly more than the control group based on outcomes measuring depression, anxiety, and global functioning. The editors of the *British Journal of Psychiatry* [54] encouraged future research to focus more on the internal mechanisms of music therapy for depression as it is known that music therapy seems to be an effective form of treatment as such. In other words, there is a need to know more specifically what in music therapy creates the effect and to identify the possible unique features of music therapy as a treatment. This task is not an easy one because the therapeutic process consists of various overlapping mechanisms and qualities that are difficult to separate from the bigger picture. In addition, music therapy practices vary in terms of clinical methods and thinking beyond the methods, one of the critiques being that interventions have not always been described in enough detail in previous studies, and that multiple methods have been employed in single studies [54]. Thus, we decided to focus only on improvisational music therapy based on IIMT [12] and try to better understand the possible unique qualities of this model. Furthermore, we wanted to investigate to what extent this model is similar or different when compared to other music therapy approaches.

One of the most important aspects of this RCT is the attention given to the interventions as such. We have carefully developed the IIMT model based on previous research results, feedback from the clinicians and clients, as well as on theoretical, methodological, and technical elaboration according to the requirements of the current trial. The fundamental aim has been to ensure the quality of the clinical work among all of the clinicians who employ the model and to finalise the IIMT model so that the communication and sharing of its key elements would be as clear as possible. Thus, the clinicians’ training before the RCT is an important, preparatory part for the later clinical work. This can be associated with the idea of treatment fidelity, whereby reliability and validity of behavioural interventions are monitored [55]. Treatment fidelity refers to the extent to which a therapist uses prescribed interventions and approaches prescribed, and avoids proscribed procedures. Furthermore, the question is about competence and skills shown by the therapist [56]. In sum, we want to ensure that the clinical work is a factor sufficiently controlled for, while at the same time acknowledging that human actions can never be fully standardised. Additionally, our aim is to take a step towards creating a standard for music therapy in the treatment of depression.

The therapeutic process of this RCT is based on 12 bi-weekly sessions of 60 min each. Although the length of the intervention is being driven by budget constraints, it is in line with current societal demands requiring treatments to be cost-effective. This means that short interventions are being used more frequently. Therefore, one of the goals of this RCT is to find out whether such a short intervention is effective, and who would benefit most in relation to variables such as depression severity and length of illness.

When a therapy process includes only 12 sessions, it is particularly crucial to ensure effective practices are employed from the very start. One of the aims of the therapists’ training is to find ways to begin the therapy process as effectively as possible, for example by developing shareable ways to quickly engage the clients. In addition to traditional music therapy-related means (such as certain musical interventions designed to create a safe and supportive relationship) we will test selected, promising methods such as listening-back homework and RFB. Both are methods that can be applied to other psychotherapy approaches as well, albeit homework in the IIMT context is very much model specific because it relies on unique aspects of the therapy model.

Listening to the clinical improvisations at home in between therapy sessions is based on our previous experience of its benefits. From the literature, we know that music stores memories and emotions in a specific way [57] and although clinical improvisations differ from composed music, which has been studied more in this context, there is anecdotal evidence about improvised music possessing similar qualities. We hypothesise that when returning to improvisations created and discussed in the music therapy sessions, one can continue therapeutic reflections by oneself and, thus, progress with certain key issues and themes between the sessions with a therapist. Homework-based work may also further stimulate and create themes for the following music therapy session, thus increasing or maximising the benefits derived from the therapy. Should this type of enhancement effect be established, then music therapy would be one of the few therapy forms where such a valuable product (music improvisations) can be stored and taken home for further processing. This is directly related to the question of the unique, beneficial qualities specific to improvisational music therapy.

A similar therapeutic enhancement can be expected from RFB, because of the shared properties between RFB and music improvisations. Indeed, both activities are able to facilitate the emergence of unexpressed themes and emotions through the creation of a non-cognitive bypass. In the case of music improvisations, this facilitation effect derives from the non-verbal and symbolic qualities of music used as an expressive medium. As for RFB, the state of calm alertness it induces appears to reduce the repression and defence mechanisms that are usually in place, thus allowing easier access to therapeutically relevant material. Consequently, it is reasonable to assume that a synergy effect might be achieved by using RFB as a prelude to improvisational music therapy.

Both LH and RFB—whose presence and absence create the study’s conditions—are particularly designed to deepen emotional processing. Results concerning the efficacy of these methods in advancing the therapy outcome will thus contribute to the broader understanding of emotional processing as an ingredient of the therapy process. This is highly important because developing the practice depends on understanding the particular roles of different active ingredients.

Overall, we believe that the clarity of the clinical technique applied in the study, the work done to achieve treatment fidelity, and the methodological strictness of the design will make the results of this RCT a valuable contribution to music therapy, and to the evidence base for the treatment of depression more generally. The endeavour to address questions about the possible unique features of music therapy and the elements responsible for the observed outcomes is a rather challenging one, yet it also bears the most relevance for music therapy as a combined science and practice.

## Trial status

At the time of manuscript submission, half of the target number of clients (34 out of 68) have finished the therapy process associated with the trial. The remaining half still need to be recruited and we expect to complete the therapy work by the end of 2018. By end of 2018 majority of the research therapies were completed and by end of March 2019 the target number of the therapy processes were accomplished. Assessments based on measurepoints 1 and 2 are going on and will last until end of 2019. We suppose to get all the outcome measure data based on measurepoints 1 and 2 typed in for the analysis during the Spring 2019.

## Abbreviations

CCT: Clinical controlled trial
CI: Confidence interval
ES: Effect size
F32: Major depressive disorder, single episode
F33: Major depressive disorder, recurrent
GAF: General functioning
HADS: Hospital Anxiety and Depression Scale
HADS-A: Anxiety subscale of the Hospital Anxiety and Depression Scale
HRVB: Heart rate variability biofeedback
ICD-10: International Statistical Classification of Diseases and Related Health Problems 10th revision
IIMT: Integrative improvisational music therapy
LH: Listening homework
MADRS: Montgomery–Åsberg Depression Scale
MCID: Minimum clinically important difference
NPNG: No Pain No Gain (name of the current trial)
RAND-36: A health survey
RCT: Randomised controlled trial
RFB: Resonance frequency breathing
SD: Standard deviation
SMD: Standardised mean difference
TAU: Treatment as usual
